# Developmental Neurotoxicity of Alcohol from Neuronal Basis to Behavioural Outcomes: A Comprehensive Review

**DOI:** 10.3390/neurolint18070123

**Published:** 2026-06-25

**Authors:** Kamal Smimih, Chaima Azzouhri, Bilal El-Mansoury, Ahmed Draoui, Hasna Lahouaoui, Abdelali Bitar, Mohamed Merzouki, Omar El Hiba

**Affiliations:** 1 Biological Engineering Laboratory, Faculty of Sciences and Technology (FST), Sultane Moulay Slimane University, Beni Mellal 23000, Morocco; kamal.smimih@usms.ma (K.S.); draouisvt@gmail.com (A.D.); m.merzouki@usms.ma (M.M.); 2 Nutritional Physiopathologies, Neuroscience and Toxicology Team, Laboratory of Anthropogenic, Biotechnology and Health, Faculty of Sciences, Chouaib Doukkali University, El Jadida 24000, Morocco; azzouhri.c823@ucd.ac.ma (C.A.); el-mansouri.b@ucd.ac.ma (B.E.-M.); bitar.a@ucd.ac.ma (A.B.); 3 Ecole Supérieure d’Education et de Formation (ESEF), Sultane Moulay Slimane University, Beni Mellal 23000, Morocco; 4 Higher Institute of Nursing Professions and Health Techniques, Ministry of Health, Laayoune 70000, Morocco; fourkane@live.fr

**Keywords:** prenatal alcohol exposure, fetal alcohol spectrum disorders, neurodevelopmental, synaptogenesis, brain

## Abstract

Prenatal alcohol exposure (PAE) is recognized as a major public health concern due to its profound and lasting effects on the central nervous system (CNS) and its ability to induce fetal alcohol spectrum disorders (FASD), which encompass a wide range of cognitive, behavioural, and neuropsychiatric disorders that persist throughout life. Experimental and clinical studies have identified several mechanisms underlying ethanol impairing brain development, including apoptosis, oxidative stress, disruption of morphogen and growth factor signalling pathways, impaired neuronal proliferation and migration, neurotransmitter systems’ dysfunction, glial cells damage associated with deficient myelination, vascular and blood–brain barrier (BBB) alterations, and lasting epigenetic reprogramming. However, to date no widely accepted integrative framework explaining how these impairments underline the heterogeneous phenotype observed in FASD is available. The present brings together developmental neurobiology and computational neuroscience to conceptualize PAE as a disorder of emerging neural and functional architecture. Here, we summarize the pharmacokinetics of ethanol in pregnancy, critical windows of vulnerability, and the classical pathways of alcohol teratogenesis affecting neuronal survival, migration, synaptogenesis, myelination, and gene regulation. We have also reviewed MRI, diffusion imaging, and EEG/MEG evidence showing altered brain volumes, white matter microstructure, functional connectivity, and network organization in individuals with PAE. Finally, we propose a systems-level model that conceptualizes PAE as a disorder of emerging neuro-computational architecture, in which ethanol-induced cellular and molecular perturbations collectively alter the building blocks and self-organization rules of brain network assembly.

## 1. Introduction

Prenatal alcohol exposure (PAE) remains one of the most common and fully preventable causes of neurodevelopmental disability worldwide [1,2]. Although many countries have introduced public health campaigns, unplanned pregnancies, cultural norms surrounding alcohol, and under-recognition of risk mean that a substantial proportion of fetuses are still exposed to alcohol during gestation [3]. The resulting clinical outcomes, grouped under the umbrella term fetal alcohol spectrum disorders (FASD), range from classic fetal alcohol syndrome (FAS) with facial dysmorphology, growth restriction, and central nervous system involvement, to more subtle alcohol-related neurodevelopmental disorders in which structural anomalies may be minimal but behavioural and cognitive impairments are pronounced [4,5].

Children and adults with FASD frequently display impairments in multiple domains: learning and memory, attention and impulse control, executive functions, language development, social cognition, motor coordination, and adaptive functioning [5,6]. These primary neurocognitive difficulties are often compounded by secondary problems such as school failure, anxiety and mood disorders, substance use, and difficulties with employment and independent living [7,8]. The phenotype is highly heterogeneous, reflecting differences in exposure patterns, timing and dose of alcohol, genetic and epigenetic background, nutritional status, and postnatal environment [9,10]. This heterogeneity complicates diagnosis and frequently leads to misdiagnosis or missed diagnosis, particularly in individuals without craniofacial features [9,10].

At the mechanistic level, experimental models in rodents, zebrafish, and non-human primates have demonstrated that ethanol interferes with key developmental processes in the brain [11,12]. Depending on the developmental stage at exposure, ethanol can impair neurulation and neural tube closure, disrupt morphogen gradients and regional patterning, reduce proliferation of neural progenitors, derail neuronal migration and cortical lamination, induce widespread apoptotic neurodegeneration during the brain growth spurt, alter synaptogenesis and dendritic arborization, impede oligodendrocyte maturation and myelination, and reprogram gene expression through oxidative stress and epigenetic modifications [13,14,15]. Human postmortem studies and neuroimaging findings are broadly consistent with these mechanisms, showing microencephaly, cortical thinning, corpus callosum agenesis or hypoplasia, cerebellar vermis hypoplasia, and diffuse white-matter abnormalities in individuals with heavy PAE [16,17].

Despite this rich mechanistic literature, a conceptual gap remains. Most studies focus on one or a few mechanisms or brain regions in isolation. Yet, the clinical expression of FASD is diffuse, multidomain, and depends critically on the coordinated operation of large-scale brain networks rather than on any single structure. In parallel, developmental neuroscience has increasingly adopted a network perspective, using graph theory and connectomics to characterize the brain as a complex system of nodes and edges that gradually organizes into efficient, modular, and hierarchically structured networks over development. This view is particularly powerful for explaining how relatively small perturbations at multiple nodes or developmental steps can accumulate to produce large systems-level effects.

The central premise of this review is that PAE is best understood not only as a collection of local injuries, but as a perturbation of the self-organizing process by which the fetal and infant brain builds its neuro-computational architecture. We argue that the various cellular and molecular insults induced by ethanol converge to shift the developmental trajectory of the brain away from typical network configurations and toward suboptimal architectures that are less efficient, less well integrated, and less robust. This perspective helps explain why the phenotype of FASD is widespread and heterogeneous, why some deficits emerge only later in childhood when the demands on network integration increase, and why interventions that broadly enhance plasticity and environmental support can be beneficial despite the absence of a single “fixable lesion”.

In the sections that follow, we first outline how ethanol is absorbed and handled in pregnancy, and why the fetus is especially vulnerable. We then review the temporal choreography of human brain development and highlight critical windows during which alcohol can interfere with neurodevelopmental processes. Next, we summarize the major mechanistic pathways of PAE and the specific aspects of brain construction they disrupt. We then turn to systems-level evidence from structural and functional neuroimaging and electrophysiology that PAE alters brain network organization. Building on this, we articulate a model of FASD as a disorder of emerging neuro-computational architecture and derive predictions and clinical implications. Finally, we identify key directions for future research needed to refine and test this framework.

## 2. Pharmacokinetics of Ethanol in Pregnancy and Fetal Exposure

Understanding how ethanol is absorbed, distributed, and eliminated during gestation is essential for interpreting its neurodevelopmental toxicity. The timing, peak intensity, and duration of fetal exposure are jointly determined by maternal pharmacokinetics, placental transfer, and the immature metabolic capacity of the fetus.

After ingestion, ethanol is rapidly absorbed along the gastrointestinal tract, primarily from the stomach and proximal small intestine [18]. Peak maternal blood alcohol concentration (BAC) depends on dose, body mass, gastric emptying, and the presence and type of food in the stomach, as well as the rate of drinking [19]. Once in circulation, ethanol disperses throughout total body water and readily crosses biological membranes, including the blood–brain barrier and the placenta, mainly by passive diffusion [18,20].

The placenta acts as a semipermeable interface rather than a protective barrier against ethanol. Experimental and clinical data show that maternal and fetal BACs equilibrate rapidly and are typically very similar, with only a short delay between maternal dosing and fetal exposure [18,21,22]. Ethanol itself is only minimally metabolized by the placenta: alcohol dehydrogenase (ADH) and cytochrome P450 2E1 (CYP2E1) are present but at low levels, and their contribution to overall clearance is small compared with maternal hepatic metabolism [23,24,25]. By contrast, the fetus has a markedly limited capacity to oxidize ethanol. The principal enzymes that mediate ethanol metabolism in adults, class I ADH and CYP2E1, are expressed at very low levels in the fetal liver and brain during early and mid-gestation and increase only toward late gestation and the early postnatal period [26,27,28]. Catalase and other alternative pathways can contribute to ethanol oxidation but are quantitatively insufficient to process the ethanol load generated by maternal drinking [25,26]. As a result, ethanol persists longer in fetal tissues than in maternal blood and is cleared predominantly by passive diffusion back across the placenta into the maternal circulation, where it is ultimately oxidized in the maternal liver [18,21]. Ethanol also partitions into the amniotic fluid. Human and animal studies show that, during the elimination phase, ethanol concentrations in amniotic fluid can exceed those in maternal and fetal blood, indicating that the amniotic cavity acts as a reservoir [21,29]. Because the fetus continually swallows amniotic fluid, ethanol present in this compartment is reintroduced into the fetal gastrointestinal tract even after maternal BAC has begun to decline [25]. This recirculation effectively prolongs fetal exposure beyond the duration of maternal intoxication and can transform brief maternal drinking episodes into extended fetal exposure windows [18,25,30]. Patterns of maternal alcohol consumption critically shape these exposure dynamics. Chronic low-to-moderate drinking produces repeated, relatively modest elevations in fetal BAC, whereas episodic binge drinking (e.g., ≥4–5 standard drinks in a short interval) generates high-peak fetal BAC and transient saturation of fetal tissues [31]. Experimental work in rodents demonstrates that such high peaks, particularly when they coincide with periods of intense neurogenesis, neuronal migration, or synaptogenesis, are especially likely to trigger widespread apoptotic neurodegeneration and interfere with early patterning processes [32,33,34]. Thus, the same average weekly alcohol dose can have very different developmental consequences depending on whether it is consumed in small, dispersed amounts or in concentrated binges [31]. Importantly, no exposure threshold has been identified below which alcohol can be considered unequivocally safe for the developing brain [35]. Large epidemiological and guideline documents converge on the conclusion that abstinence during pregnancy is the only evidence-based recommendation, given substantial uncertainty around individual susceptibility and the absence of randomized safety trials [20,36]. Susceptibility varies widely between pregnancies and is shaped by maternal metabolic capacity, placental function, genetic polymorphisms in alcohol- and aldehyde-metabolizing enzymes (e.g., ADH1B, ALDH2, CYP2E1), nutritional status (including folate and choline availability), and co-exposures such as tobacco, other drugs, infection, and psychosocial stress [37,38,39]. From a mechanistic standpoint, even a single binge episode overlapping a critical period of brain development may be sufficient to induce long-lasting alterations in neural circuitry, particularly in regions undergoing intense proliferation, migration, or synaptic refinement at that time [33].

## 3. Mechanistic Actions of Prenatal Alcohol on the Developing Brain

Within the developmental framework outlined above, PAE can be conceptualized as a set of mechanistic perturbations acting across multiple biological scales. Ethanol engages partially overlapping injury cascades that affect neuronal survival, redox homeostasis, morphogen and growth-factor signalling, neurotransmission, glial function, and epigenetic regulation [40].

A substantial portion of the mechanistic evidence derives from experimental animal models, which provide important causal insights but require cautious interpretation due to species differences in developmental timing and brain maturation.

### 3.1. Ethanol-Induced Apoptotic Neurodegeneration

One of the most robust observations from animal models is that acute, high-dose ethanol exposure during the brain growth spurt triggers widespread, synchronous apoptotic neurodegeneration [41]. In rodents, a single binge-like exposure around postnatal day 7—a period of intense synaptogenesis considered broadly analogous to late third-trimester development in humans—induces caspase-3 activation and TUNEL-positive profiles across the cortex, hippocampus, thalamus, and cerebellum [32,33,42].

The developmental switch of GABAergic signalling from depolarizing to hyperpolarizing represents a critical maturational event that may contribute to periods of increased vulnerability during early brain development. In the immature brain, ethanol behaves as a positive allosteric modulator of GABA-A receptors and as an NMDA receptor antagonist [14]. The survival of developing neurons depends on patterned NMDA-mediated calcium influx and activity-dependent trophic signalling [43]. Abrupt NMDA blockade combined with excessive GABAergic inhibition mimics loss of necessary excitatory drive, promoting pro-apoptotic cascades (e.g., Bax activation, cytochrome c release, caspase activation) in neurons that had been integrating into nascent circuits [33,44]. These findings are robust in animal models, although their direct quantitative translation to human development remains limited.

The vulnerability window is sharply delimited: exposures outside peak synaptogenesis are markedly less apoptogenic, underscoring the critical-period nature of this effect [32,45].

**Figure 1:**
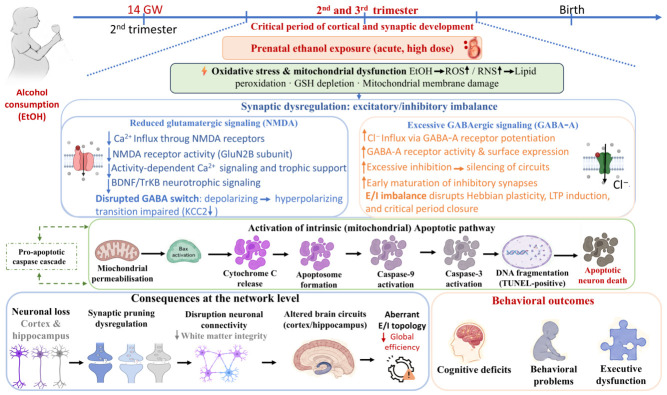
Schematic overview of the cellular and network-level mechanisms underlying prenatal ethanol exposure (PAE) induced neurotoxicity and associated behavioural outcomes. The timeline at the top indicates the gestational windows of vulnerability, with the second and third trimesters corresponding to the critical period of cortical and synaptic development. Oxidative stress band: ETOH-induced reactive oxygen species (ROS) and reactive nitrogen species (RNS) amplify mitochondrial membrane damage, glutathione (GSH) depletion, and lipid peroxidation, converging with E/I imbalance to trigger pro-apoptotic signalling. Synaptic dysregulation panel: Ethanol exerts dual opposing effects on synaptic signalling, suppressing NMDA receptor-mediated glutamatergic transmission (reduced Ca^2+^ influx, ↓BDNF/TrKB signalling, and impaired KCC2-dependent GABA depolarizing-to-hyperpolarizing switch) while simultaneously potentiating GABA-A receptor-mediated inhibitory currents, leading to excitatory/inhibitory (E/I) imbalance and disrupted Hebbian plasticity. Middle panel: The intrinsic (mitochondrial) apoptotic pathway is activated sequentially: pro-apoptotic signals promote mitochondrial outer membrane permeabilization (MOMP)via Bax/Bak activation, cytochrome c release into the cytosol, apoptosome assembly (cytochrome c + Apaf-1 + dATP), and sequential activation of caspase-9 and caspase-3, culminating in DNA fragmentation (TUNEL-positive cells) and irreversible neuronal death. Bottom left panel: At the network level, neuronal loss triggers a cascade of structural and functional alterations, including dysregulated synaptic pruning, decreased white matter integrity, disrupted long-range connectivity, and reduced global network efficiency with aberrant E/I topology, collectively impairing the emergence of functional brain architecture. Bottom right panel: These neurodevelopmental impairments manifest as the heterogeneous behavioural phenotype of fetal alcohol spectrum disorders (FASD), encompassing cognitive deficits, behavioural problems (hyperactivity, impulsivity), executive dysfunction, and neuropsychiatric features including anxiety, depression, and ASD-like traits. PAE, prenatal alcohol exposure; EtOH, ethanol; NMDA, N-methyl-D-aspartate; GABA, gamma-aminobutyric acid; BDNF, brain-derived neurotrophic factor; KCC2, potassium-chloride cotransporter 2; ROS, reactive oxygen species; RNS, reactive nitrogen species; GSH, glutathione; MOMP, mitochondrial outer membrane permeabilization; Apaf-1, apoptotic protease activating factor-1; TUNEL, terminal deoxynucleotidyl transferase dUTP nick end labelling; E/I, excitatory/inhibitory; FASD, fetal alcohol spectrum disorder; ASD, autism spectrum disorder.

The anatomical consequences are long-lasting. Selective neuron loss reduces regional volumes and neuron density, often in layers or nuclei undergoing intense synaptogenesis at the time of exposure [46]. Purkinje cells in cerebellar cortex and pyramidal neurons in hippocampal CA1 are particularly sensitive, leading to enduring changes in motor coordination and memory-related circuitry even when gross morphology appears relatively preserved [32,47]. Beyond overt waves of cell death, more moderate or repeated exposures may subtly bias the balance between physiological programmed cell death and survival, skewing pruning trajectories toward either excessive elimination of synapses or aberrant retention of atypical connection search with distinct implications for later network topology [45] (Figure 1).

### 3.2. Oxidative and Nitrosative Stress, Mitochondrial Injury, and Energy Failure

Ethanol metabolism generates acetaldehyde and reactive oxygen species (ROS), including superoxide, hydrogen peroxide, and hydroxyl radicals [48]. In parallel, ethanol can enhance production of reactive nitrogen species (RNS) via the up-regulation of nitric oxide synthases and formation of peroxy nitrite [49]. In neurons and glia, these processes engage NADPH oxidase, xanthine oxidase, and mitochondrial electron transport chain components as both sources and targets of oxidative injury [50].

The fetal and early postnatal brain is particularly vulnerable because antioxidant systems (superoxide dismutase, catalase, glutathione peroxidase) are immature, and glutathione stores are limited [51]. ROS and RNS damage membrane lipids, proteins, and nucleic acids [51,52]. Lipid peroxidation is especially detrimental in the developing CNS, where myelin and neuronal membranes are enriched in polyunsaturated fatty acids [51,53]. Oxidative DNA damage can trigger cell-cycle arrest, apoptosis, or mutagenesis, while protein oxidation alters receptor function, ion channel gating, and cytoskeletal dynamics [50,54].

Mitochondria occupy a central position in this cascade. Ethanol and acetaldehyde can depolarize mitochondrial membranes, inhibit respiratory chain complexes, increase ROS leakage, and reduce ATP production [55]. As a result, developing neurons and oligodendrocyte progenitors face an energetic shortfall precisely when they require high ATP levels for neurite outgrowth, synaptogenesis, and myelination [56]. Impaired mitochondrial calcium buffering further sensitizes cells to excitotoxic and apoptotic insults. Experimental studies in immature neurons support a causal role for oxidative stress: pharmacological antioxidants and mitochondria-targeted scavengers can partially attenuate ethanol-induced neurodegeneration, although they do not fully normalize structural and functional outcomes [50,57].

### 3.3. Disruption of Morphogen and Growth Factor Signalling

Ethanol also interferes with the signalling pathways that orchestrate early patterning and later neuronal differentiation [13,58]. The Sonic hedgehog (Shh) pathway is essential for ventral neural tube patterning and midline brain and facial development [59,60]. Gene environment interaction models demonstrate that transient ethanol exposure during neurulation interacts with partial deficits in Shh pathway components (e.g., the coreceptor Cdon) to produce holoprosencephaly-like phenotypes, including midline facial anomalies and forebrain hypoplasia [61]. In avian and zebrafish models, ethanol reduces Shh expression in promigratory neural crest and rostroventral neural tube and synergizes with pharmacologically smoothened antagonists or planar-cell polarity mutations to exacerbate craniofacial defects [62].

Retinoic acid (RA), derived from vitamin A, is another morphogen implicated in ethanol teratogenicity [63]. Ethanol oxidation can compete with retinaldehyde metabolism, perturb RA synthesis, and alter RA dependent anterior–posterior patterning [64]. Disrupted gradients of Shh and RA during early gestation may lead to aberrant regional specification and altered balance between proliferation and differentiation in progenitor zones, providing a mechanistic link between early exposures and midline or regional brain abnormalities observed in FASD [62,65].

Later in gestation, neurotrophins such as brain-derived neurotrophic factor (BDNF), nerve growth factor (NGF), and insulin-like growth factor-1 (IGF-1) support neuronal survival, dendritic growth, synaptogenesis, and plasticity [66]. Prenatal ethanol decreases expression of neurotrophins and their receptors in multiple brain regions and alters downstream signalling through ERK/MAPK and PI3K/Akt pathways, thereby compromising maturation and stabilization of synaptic networks [67,68].

**Figure 2:**
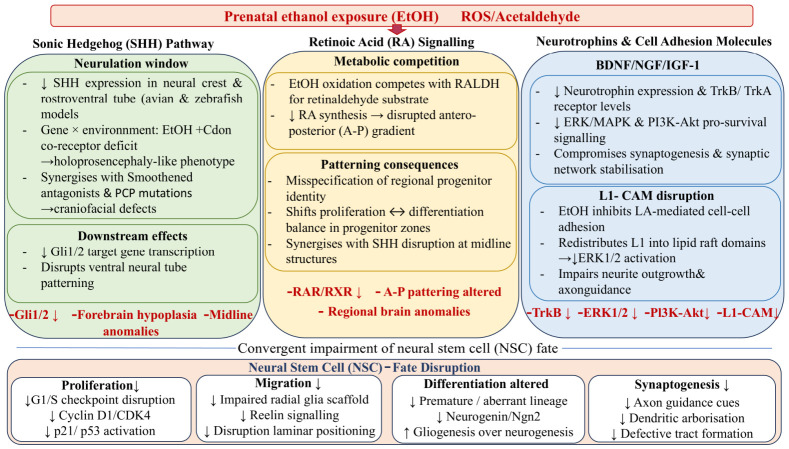
Conceptual illustration of ethanol-induced disruption of morphogen and growth factor signalling during CNS development. Speculative pathways are explicitly labelled and should not be interpreted as established facts. PAE generates ROS, RNS, and acetaldehyde acting on three major developmental signalling axes. (1) SHH pathway: EtOH reduces SHH in neural crest and rostroventral tube; synergizes with Cdon deficit to produce holoprosencephaly-like phenotypes. Downstream Gli1/Gli2 suppression is model-based. (2) RA signalling: EtOH competes with RALDH for retinaldehyde, reducing RA synthesis and disrupting A-P patterning; downstream misspecification of progenitor identity is speculative. (3) Neurotrophin and L1-CAM: PAE decreases BDNF, NGF, IGF-1 and TrkB/TrkA, attenuating ERK/MAPK and PI3K-Akt cascades. EtOH inhibits L1-CAM adhesion, redistributes L1 into lipid rafts, and suppresses ERK1/2, impairing neurite outgrowth. These pathways converge on NSC fate, impairing proliferation, migration, differentiation, and synaptogenesis, producing the structural and functional FASD phenotype. SHH, Sonic hedgehog; Cdon, cell adhesion molecule-related/down-regulated by oncogenes; PCP, planar cell polarity; RA, retinoic acid; RALDH, retinaldehyde dehydrogenase; A-P, antero–posterior; BDNF, brain derived neurotrophic factor; NGF, nerve growth factor; IGF-1, insulin-like growth factor-1; TrkB/A, tropomyosin receptor kinase B/A; ERK, extracellular signal-regulated kinase; MAPK, mitogen-activated protein kinase; PI3K, phosphoinositide 3-kinase; L1-CAM, L1 cell adhesion molecule; NSC, neural stem cell; LTP, long-term potentiation; E/I, excitatory/inhibitory; FASD, fetal alcohol spectrum disorders.

Cell adhesion molecules (CAMs) and extracellular matrix components are also highly ethanol sensitive [69]. The L1 cell adhesion molecule is a particularly well studied target: ethanol at concentrations reached during social drinking potently inhibits L1-mediated cell–cell adhesion, redistributes L1 into lipid raft domains, and disrupts L1-dependent activation of ERK1/2, thereby impairing neurite outgrowth and axon guidance [70]. Given that mutations in L1 reproduce many neuroanatomical abnormalities seen in FAS (hydrocephalus, callosal agenesis, cerebellar dysplasia), disruption of L1 and related CAMs provides a plausible route by which ethanol perturbs neuronal migration, tract formation, and synaptogenesis, even in the absence of gross malformations [47,61] (Figure 2).

### 3.4. Neurotransmitter System Alterations and Activity-Dependent Mis-Programming

Beyond its acute effects on NMDA and GABA-A receptors, repeated PAE produces enduring changes in neurotransmitter systems [71]. Animal models show compensatory up-regulation of NMDA receptor subunits, shifts in NR2A/NR2B ratios, altered GABA-A receptor subunit composition, and modifications in glutamate and GABA transporter expression [44,68]. Monoaminergic systems (dopamine, serotonin, noradrenaline) are also affected at the level of neuron number, axonal innervation density, and receptor/transporter expression, particularly in meso–cortico–limbic and frontostriatal circuits [72,73].

These changes matter because neurotransmitters in the developing brain act not only as fast synaptic signals but also as trophic and instructive cues [74]. Spontaneous and sensory-driven activity patterns, early network oscillations, spindle bursts, and immature theta and gamma rhythms shape synaptic refinement and receptive-field formation [75]. If ethanol alters intrinsic excitability, synaptic gain, or the temporal structure of these patterns, then Hebbian and spike timing-dependent plasticity rules will be applied to an abnormal activity landscape [76]. Synapses that would normally be stabilized can be weakened or eliminated, whereas atypical connections may be inappropriately strengthened [44,68].

Human neurophysiology findings are consistent with this view. EEG and MEG studies in infants and children with PAE or FASD reveal altered resting-state spectral power (including early hypersynchrony followed by reduced alpha power), abnormal alpha peak frequency, and disrupted long-range oscillatory coupling, sometimes even in the absence of facial dysmorphology [77,78]. These changes point to a persistent disturbance in excitation–inhibition balance and in the large-scale temporal coordination that underlies efficient information processing.

### 3.5. Glial Injury, Neuroinflammation, and White-Matter Abnormalities

Glial cells are central architects and maintainers of neural circuits and are therefore key targets of PAE [79]. Astrocytes regulate extracellular potassium and glutamate, provide metabolic support through lactate shuttling, secrete synaptogenic proteins (e.g., thrombospondins, hevin), and contribute to synaptic pruning [80,81]. Ethanol impairs astrocyte proliferation, alters expression of glutamate transporters (e.g., GLT-1/EAAT2, GLAST/EAAT1), and modifies cytokine and growth factor secretion profiles, with downstream impacts on neuronal survival and synapse formation [47,82].

Oligodendrocyte progenitors and immature oligodendrocytes are particularly vulnerable to oxidative stress and excitotoxic insults [83]. Experimental models of PAE demonstrate reduced oligodendrocyte numbers and delayed or incomplete myelination in multiple tracts [47,84].

Concordantly, diffusion tensor imaging in children with FASD reveals reduced fractional anisotropy and increased radial diffusivity in major white matter pathways, including the corpus callosum, superior longitudinal fasciculus, internal capsule, cingulum, and cerebellar peduncles, even in children without the full physical stigmata of FAS [85,86]. These microstructural abnormalities likely reflect a combination of reduced axon packing density, altered axon calibre, and hypomyelination, and they correlate with cognitive and behavioural impairments.

Microglia, the resident immune cells of the CNS, are instrumental in synaptic pruning and in responding to developmental insults [87]. Binge-like ethanol exposure in early postnatal mice induces the rapid activation of microglia and longer-lasting changes in astrocytic reactivity [88]. PAE can “prime” microglia toward a pro-inflammatory state, characterized by increased production of cytokines and altered phagocytic activity, such that subsequent challenges (infection, stress, additional toxins) elicit exaggerated responses [47]. Persistent low-grade neuroinflammation in the developing brain may exacerbate oxidative damage, further injure oligodendrocytes, and derail the finely tuned balance of synaptic pruning, contributing to region-specific over or under pruning of circuits [45,82].

### 3.6. Epigenetic Reprogramming and Potential Transgenerational Effects

Epigenetic mechanisms offer a molecular substrate for durable changes in gene expression following transient exposures [89]. In rodent models and human bio samples, PAE is associated with altered DNA methylation at promoters and regulatory elements, changes in histone acetylation and methylation, and dysregulation of microRNAs that control networks of genes involved in neurogenesis, synaptic plasticity, endocrine signalling, and stress responses [90,91,92].

For example, PAE can modify methylation and expression of genes in the HPA axis, the serotonin system, and the synaptic scaffolding pathways, with effects persisting into adolescence and adulthood [93,94]. Some epigenetic alterations have been observed in germ cells and in offspring of exposed animals, suggesting that at least part of the PAE signal can be transmitted to the next generation, although the extent and relevance of such transgenerational effects in humans remain under active investigation [95].

At the level of developing neural circuits, aberrant epigenetic programming can shift developmental trajectories by altering the timing, amplitude, and cell-type specificity of gene expression programmes that govern neuronal differentiation, synapse formation, and plasticity [92]. In this sense, PAE does not merely injure existing cells; it also modifies the “rules” by which networks self-organize and adapt, providing a molecular bridge between transient exposure and long-lasting reconfiguration of neuro-computational architecture [91,96]. However, most evidence remains derived from animal and experimental models, and the exact contribution of these mechanisms to human outcomes requires further validation.

## 4. Systems-Level Evidence for Altered Network Development in PAE

Mechanistic disruptions at the cellular and circuit level are expected to scale up into measurable alterations in brain architecture and connectivity. Structural and diffusion MRI, functional MRI, and electrophysiological studies converge on the view that PAE reshapes the development of large-scale networks, rather than simply causing diffuse atrophy or focal lesions [86,97,98,99].

### 4.1. Structural MRI: Regional Volumetric Changes

Quantitative structural MRI consistently shows reduced intracranial volume and regional grey matter reductions in children and adolescents with FASD [100]. Early work already demonstrated prominent volume loss in frontal regions, basal ganglia, corpus callosum, and cerebellar structures [101,102]; more recent analyses confirm that the hippocampus and caudate are particularly affected in youth with FASD [97]. Even after adjusting for intracranial volume using normative modelling, subcortical structures such as bilateral hippocampi, putamen, and pallidum remain disproportionately small, indicating selective vulnerability rather than a uniform scaling-down of the brain [97].

Cortical thickness and surface area analyses reveal complex, regionally specific patterns: thinning is frequently reported in frontal and temporal association cortices, with relative sparing of primary sensory regions [98,99]. These anatomical profiles align with the executive, memory, and social cognition impairments that characterize FASD.

Corpus callosum abnormalities are particularly striking. Agenesis or hypogenesis, segmental thinning, and shape anomalies of the callosal body, genu, and splenium are frequently reported in both youth and adults with FASD [101,103]. Midline anomalies of this kind likely reflect a combination of early patterning defects, impaired axon guidance at the midline, and selective apoptosis of callosal projection neurons, and they have been linked behaviourally to deficits in response inhibition and cognitive flexibility [98].

### 4.2. Diffusion MRI: White-Matter Microstructure and the Connectome Backbone

Diffusion tensor imaging and more advanced diffusion models provide complementary insight into white-matter architecture. Across more than twenty DTI studies, individuals with PAE show widespread reductions in fractional anisotropy and increases in mean and radial diffusivity in major commissural, association, and projection pathways [86,104]. The corpus callosum, especially the genu and splenium superior and inferior longitudinal fasciculi, cingulum bundle, cerebellar peduncles, internal capsule, and corticospinal tracts, is among the most consistently affected structures [104,105,106,107].

Microstructural callosal alterations are associated with poorer response inhibition and eye-movement control, directly linking white-matter integrity to executive dysfunction [108]. Longitudinal work suggests that atypical trajectories of white-matter development persist into childhood, with combined prenatal alcohol and tobacco exposure further altering microstructural maturation [109].

Connectome reconstructions using diffusion data and graph theory show that these tract-level abnormalities translate into systems-level changes [110]. Children with PAE exhibit altered structural brain network organization, with reduced global efficiency and changes in clustering and modular structure, particularly within cortico–basal ganglia–thalamo cortical modules [111]. In early childhood, PAE-related differences in structural connectivity within language-related networks have been linked to poorer language outcomes [112]. However, connectomic findings in PAE remain sensitive to methodological choices that limit cross-study comparability, including tractography reconstruction method, parcellation atlas, and graph-theoretical thresholding approach [111]. Sample sizes in pediatric PAE connectomic studies are frequently small, and findings can diverge across developmental stages, such that structural connectome alterations reported in neonates do not necessarily mirror those reported in school-age children and adolescents, suggesting that network-level effects of PAE may not generalize uniformly across age [111].

### 4.3. Functional Connectivity: Resting and Task-Based Networks

Resting state fMRI studies show that PAE is associated with alterations in intrinsic functional network organization [113]. In school-aged children with FASD, low-frequency BOLD correlations reveal reduced within-network connectivity in the default mode, salience, and attention networks, alongside atypical cross-network coupling [107,114]. In adults with fetal alcohol syndrome, cognition-related networks, including the default mode and executive control networks, show disrupted connectivity patterns that relate to neuropsychological performance [107].

A recent study in 6–8-year-old children with FASD demonstrated reduced functional connectivity between the medial prefrontal cortex and limbic regions (amygdala, hippocampus, brainstem) within the default mode network, as well as altered connectivity within the fronto–parietal network; these connectivity changes correlated with sustained attention and response inhibition deficits [115]. In neonates with PAE, combined structural functional network analyses indicate that hubs are already biased toward temporal and limbic regions rather than forming the more distributed hub layout observed in unexposed infants [111]. This suggests that network maturation is misdirected from the earliest stages, before behavioural symptoms can be clinically appreciated. Findings across resting-state functional connectivity studies in PAE are not always convergent in direction or location: reported alterations range from localized reductions in specific connections [114] to broader network-level changes affecting sensorimotor systems [113], and replication across independent cohorts using harmonized acquisition and analysis pipelines remains limited.

Task-based fMRI studies converge on the picture of inefficient and compensatory network recruitment. During spatial working memory, response inhibition, and executive tasks, children with PAE typically show hypoactivation of canonical fronto–parietal and cingulo–opercular control regions, coupled with hyperactivation in auxiliary cortical or subcortical regions, often in the setting of reduced performance [116,117]. These patterns are consistent with a system that must over-recruit additional regions to achieve a given level of performance, reflecting suboptimal wiring and altered cost efficiency trade-offs at the network level.

### 4.4. Electrophysiology: Oscillations, Coherence, and Neural Noise

EEG and MEG studies provide temporally precise evidence that PAE affects the dynamic coordination of neural activity [118]. Infants and children with PAE or FASD often show atypical resting state spectral profiles, including reduced alpha peak frequency, reduced alpha power, and alterations in theta and beta bands; such changes have been linked to deficits in visual attention and cognitive performance [119]. MEG studies indicate disturbed visuo-cortical network dynamics and delayed sensory processing in preschool and school-aged children with FASD, suggesting that basic sensory perceptual timing is already altered [77,78].

Connectivity metrics derived from EEG/MEG, such as coherence, phase-locking, and graph-based measures, tend to show reduced long-range coupling and altered hub structure, particularly in networks supporting attention and cognitive control [115]. Event-related potential studies complement these findings: in Go/No Go paradigms and other cognitive tasks, children with heavy PAE show delayed and reduced ERP components associated with stimulus evaluation and response inhibition, as well as increased trial-to-trial variability in ERP amplitude and latency, consistent with heightened neural noise and reduced stability of network states [119,120]. Nonetheless, EEG/MEG connectivity findings in PAE vary considerably across studies in the specific frequency bands, regions, and connectivity metrics implicated, and few findings have been independently replicated in separate cohorts using harmonized acquisition and analysis pipelines [118,119].

## 5. Conceptualizing PAE as a Disorder of Emerging Neuro-Computational Architecture

The convergence of cellular, glial, and epigenetic mechanisms with imaging and electrophysiological findings supports a shift in perspective: FASD are not only “lesions of particular brain regions” but disorders of how the brain’s computational architecture emerges over development [47]. The developing brain can be framed as a complex adaptive network in which nodes (neurons, columns, regions) and edges (synapses, tracts, functional couplings) self-organize into an arrangement that balances efficiency, robustness, and flexibility under biophysical constraints [121,122,123]. This self-organization is canalized by genetic and molecular programmes, yet is continuously shaped by experience, spontaneous activity, and metabolic constraints [124].

In typical development, structural and functional networks gradually converge on several canonical properties. Graph theoretical analyses show a combination of high global efficiency (short path lengths enabling rapid information transfer) and high local efficiency and clustering that support specialized processing within modules [123,125]. Modularity becomes more clearly differentiated across childhood and adolescence, with communities corresponding to sensory, motor, default-mode, and control networks [126,127]. High-degree “rich-club” hubs in association cortices and subcortical relay structures form a densely interconnected backbone that supports integration across distributed modules [125,128].

More recent manifold and gradient approaches indicate that this architecture is organized along a sensorimotor association axis: primary sensorimotor and visual regions occupy one end of the hierarchy, while trans modal association cortices (default-mode and fronto–parietal regions) occupy the other [129,130]. These large-scale gradients are already detectable in late gestation and early infancy, further differentiate during childhood, and are tightly coupled to cognitive outcomes [127]. Across this developmental window, networks become more segregated within modules yet more efficient globally, and hub regions gradually consolidate in the heteromodal association cortex [128,131].

**Figure 3:**
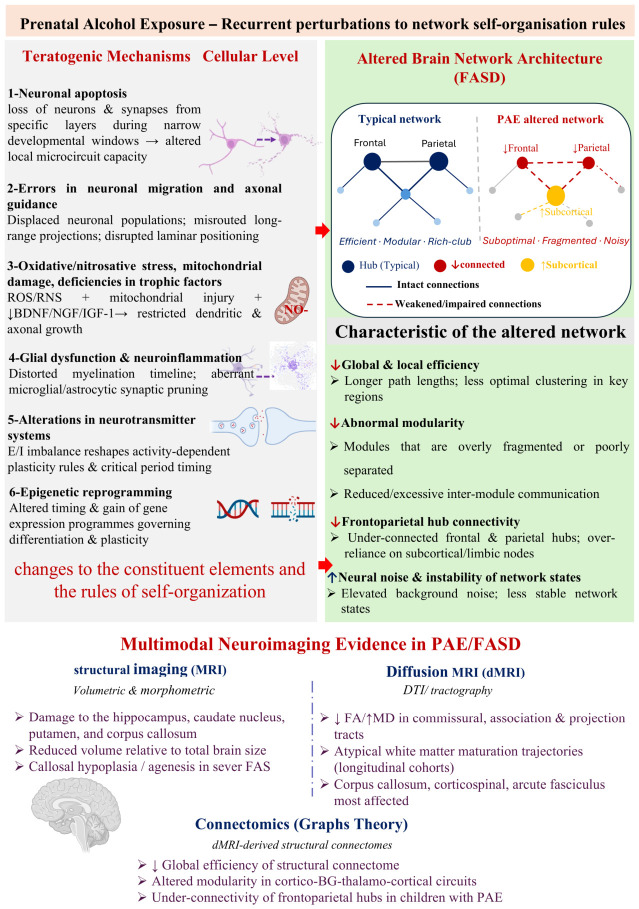
Conceptual illustration of PAE as a disorder of emerging neurocomputational architecture. Speculative inferences are explicitly indicated. Left panel: Six experimentally supported teratogenic mechanisms disrupt the building blocks of brain development: neuronal apoptosis; axonal migration and orientation errors; oxidative/nitrosative stress and deficits in trophic factors; glial dysfunction and neuroinflammation; neurotransmitter E/I imbalance; and epigenetic reprogramming. Right panel: These mechanisms converge to steer the developing connectome toward a suboptimal configuration characterized by reduced global and local efficiency, abnormal modularity, under-connected fronto–parietal hubs, excessive dependence on subcortical nodes, and high neuronal noise. The attractor landscape framework is speculative. Neuroimaging panel: Structural MRI reveals disproportionate volumetric reductions in the hippocampus, caudate nucleus, putamen, and corpus callosum; diffusion MRI documents persistent microstructural abnormalities in white matter and atypical maturation trajectories; connectomic analyses show reduced global efficiency and an altered modular structure with under-connected fronto–parietal nodes; fMRI, EEG, and MEG provide converging evidence of disrupted functional networks and altered oscillatory coherence. PAE, prenatal alcohol exposure; FASD, fetal alcohol spectrum disorders; ROS, reactive oxygen species; RNS, reactive nitrogen species; BDNF, brain-derived neurotrophic factor; NGF, nerve growth factor; IGF-1, insulin-like growth factor 1; E/I, excitatory/inhibitory; MRI, magnetic resonance imaging; DTI, diffusion tensor imaging; FA, fractional anisotropy; MD, mean diffusivity; BG, basal ganglia; DMN, default mode network; EEG, electroencephalography; MEG, magnetoencephalography; fMRI, functional MRI.

Within this framework, PAE can be viewed as a set of recurrent perturbations to the rules governing how this architecture is assembled (Figure 3) [132]. Mechanistically, ethanol-induced apoptosis removes neurons and synapses from specific layers and nuclei during narrow developmental windows, altering local microcircuit capacity [32,33]; migration and axon-guidance errors misplace neuronal populations and misroute long-range projections [133]; oxidative and nitrosative stress, mitochondrial injury, and trophic factor deficits restrict dendritic and axonal growth [134]; glial dysfunction and neuroinflammation distort myelination and synaptic pruning [135]; shifts in neurotransmitter systems reshape activity patterns that instruct plasticity [136]; and epigenetic reprogramming modifies the timing and gain of gene expression programmes that underlie differentiation and plasticity [137]. Together, these processes alter both the available “building blocks” and the activity-dependent rules that drive network self-organization (Figure 3) [138].

Systems-level evidence broadly supports this conceptual framework. Structural and diffusion MRI studies consistently demonstrate abnormalities involving the corpus callosum, subcortical structures, and major white-matter pathways, while graph-theoretical analyses reveal altered network efficiency and hub organization in children with PAE [139,140]. Functional neuroimaging and electrophysiological studies further indicate disrupted intrinsic connectivity and atypical oscillatory dynamics, suggesting that PAE affects both the structural backbone and functional coordination of large-scale brain systems [77,141,142].

From a complex systems perspective, these findings suggest that PAE biases the developing brain away from the typical attractor basins in the space of possible architectures [141]. Instead of converging on a highly efficient, modular, rich-club network with association–cortex hubs along a mature sensorimotor association axis [142], the PAE-affected brain may stabilize in suboptimal configurations characterized by: (i) reduced global and local efficiency (longer path lengths and less optimal clustering in key regions) [111]; (ii) abnormal modular structure, ranging from overly fragmented modules with weak inter-module communication to poorly segregated modules with excessive crosstalk [143]; (iii) atypical hub distribution, with under-connected frontal and parietal hubs and over-reliance on subcortical or limbic hubs that support more rudimentary control policies [144]; and (iv) elevated background neural noise and less stable network states, making it harder to maintain coherent activation patterns over time [77,123,141].

Clinically, such architectures need not produce catastrophic loss of a single function. Rather, they degrade performance across multiple domains that depend on long-range integration and flexible coordination, executive control, working memory, complex language, adaptive social cognition, and multi-step learning, producing the broad pattern of “cognitive dysmaturity” described in FASD cohorts [98]. Behavioural heterogeneity can then be understood as reflecting which networks and developmental windows were most affected and how plastic compensatory mechanisms reconfigured the remaining architecture [145].

This perspective also naturally accommodates individual variability. The impact of PAE on emerging neuro-computational architecture depends on the timing, dose, and pattern of exposure, as well as on genetic and environmental moderators that shape resilience and plasticity [38,146,147]. Early, heavy exposure coinciding with callosal development and midline patterning may yield pronounced interhemispheric dysconnection and visuomotor integration deficits; later exposures during fronto–parietal myelination and hub consolidation may preferentially compromise executive functions and goal-directed control [103]. Normative modelling approaches already show that some children with FASD exhibit extreme deviations in subcortical or callosal structure, whereas others show milder, distributed anomalies [140,147]. Within the proposed framework, these are not separate “subtypes” so much as different points in a space of altered architectures, all characterized by a shared theme: a brain that has settled into a non-optimal configuration for distributed computation as a consequence of PAE.

**Figure 4:**
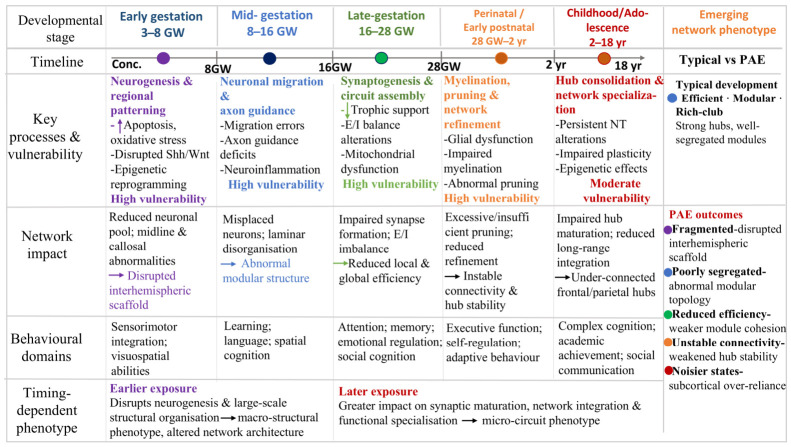
Critical windows of vulnerability: Timing of prenatal alcohol exposure (PAE) shapes emerging neuro-computational architecture. Timeline panel: Five partially overlapping neurodevelopmental processes are differentially vulnerable to PAE across gestational and postnatal stages: neurogenesis and regional patterning (3–8 GW); neuronal migration and axon guidance (8–16 GW); synaptogenesis and circuit assembly (16–28 GW); myelination, synaptic pruning, and network refinement (28 GW–2 yr); and hub consolidation and network specialization (2–18 yr). All five windows carry high vulnerability to PAE, with moderate vulnerability persisting into childhood and adolescence. Key processes panel: Dominant teratogenic mechanisms shift across windows from apoptosis, oxidative stress, and disruption of Shh/Wnt patterning signals in early gestation, to migration errors and neuroinflammation in mid-gestation, E/I imbalance and mitochondrial dysfunction during late gestation, glial dysfunction and abnormal pruning perinatally, and persistent neurotransmitter alterations and impaired plasticity in childhood. Network impact panel: Each window produces stage-specific architectural consequences, ranging from disrupted interhemispheric scaffold and abnormal modular structure in early exposures, to reduced local and global efficiency, unstable connectivity, and under-connected frontal and parietal hubs following later exposures. Timing-dependent phenotype: Earlier exposures preferentially disrupt neurogenesis and large-scale structural organization, yielding a macro-structural phenotype; later exposures exert greater impact on synaptic maturation, network integration, and functional specialization, yielding a micro-circuit phenotype. Emerging network phenotype panel: Typical development produces an efficient, modular, rich-club architecture with strong hubs and well-segregated modules; PAE steers the connectome toward one of several suboptimal configurations, including fragmented connectivity, poorly segregated modules, reduced efficiency, unstable hub dynamics, and noisier network states, depending on the timing and severity of exposure. PAE, prenatal alcohol exposure; GW, gestational weeks; E/I, excitatory/inhibitory ratio; NT, neurotransmitter; Shh, Sonic hedgehog; Wnt, Wingless-related integration site; yr, year(s); Conc., conception.

Notably, important windows of vulnerability should be taken into consideration when interpreting these varied results. Neuronal proliferation and apoptosis, neuronal migration and axonal guidance, synaptogenesis, myelination, and activity-dependent circuit refinement are among the neurodevelopmental processes impacted by PAE that take place during certain and partially overlapping developmental phases [148]. As a result, the timing of alcohol exposure plays a significant role in determining the phenotype that results. While later exposures have a greater impact on synaptic maturation, network integration, and functional specialization, earlier insults preferentially disrupt neurogenesis and large-scale structural organization [149]. The mechanical cascade depicted in Figure 3 gains a significant layer of specificity from this temporal dimension, which is further described in Figure 4, which maps developmental windows onto the creation of large-scale brain network architecture.

## 6. Mechanistic Consequences and Testable Predictions

At the structural level, diffusion MRI-derived connectomes in individuals with PAE are predicted to show reduced global efficiency and altered hub profiles relative to age-matched controls [111]. High degree nodes in fronto–parietal and cingulo–opercular networks may be less connected and less central, whereas hubs in limbic or subcortical territories may be relatively preserved or even transiently over-central early in life, consistent with existing reports of disrupted rich-club organization and atypical nodal centrality in PAE cohorts [111]. These deviations are expected to align with tract-level abnormalities described in diffusion studies, particularly in the corpus callosum, association tracts, and corticobasal ganglia circuits [99,146].

Functional networks measured with resting-state fMRI or EEG/MEG may likewise exhibit altered modular organization and an increased “cost of integration” [150]. In children and adults with FASD, default mode, executive, salience, and dorsal attention networks show reduced within-network coherence and atypical cross-network coupling, suggesting weakened segregation and inefficient integration [107,149].

These structural and functional findings are broadly convergent across imaging modalities, although variability in methodologies and cohort characteristics should be considered when interpreting cross-study consistency. For instance, structural connectome findings in PAE have varied depending on age range and cohort: alterations reported in neonatal connectomes are not necessarily of the same direction or magnitude as those reported in children and adolescents [111], and cross-cultural cohort studies have not always replicated the same network-level associations with cognitive outcomes [112]. This suggests that the connectomic signature of PAE, if it exists, may be developmentally dynamic rather than a fixed alteration detectable with a single network metric at any age.

Within this model, one expects under-segregated higher-order networks with less flexible coupling, alongside relative intrusion of sensorimotor or limbic networks into association territories patterns that have begun to emerge in recent studies of children with PAE and FASD [116,119].

If PAE primarily alters network architecture, graph theoretical metrics may covary with cognitive and behavioural outcomes within PAE cohorts [151]. Individuals showing greater reductions in global efficiency, more atypical hub centrality in the fronto–parietal cortex, or more abnormal callosal connectivity are often reported to exhibit more severe deficits in processing speed, working memory, executive control, and adaptive functioning [116,119]. Conversely, children whose connectomes remain closer to typical norms, despite confirmed exposure, may show comparatively preserved performance, consistent with normative-modelling results in PAE samples [152].

The model also predicts an age-dependent unfolding of impairments. Early in life, when behaviour depends heavily on local sensory and sensorimotor networks, global milestones may appear only mildly delayed [153]. As development increasingly relies on long-range integration across association cortices during school age and adolescence, deficits in complex reasoning, abstract language, and social cognition may become more evident and diverge progressively from those of typically developing peers [98]. Longitudinal work has shown widening gaps in executive and academic outcomes in children with PAE, particularly in domains that depend on distributed fronto–parietal and fronto–striatal circuitry [154].

A further implication is that individuals with documented PAE who do not meet full diagnostic criteria for FASD may still show subtle network alterations detectable with sensitive structural and functional measures [155]. Under high cognitive load or stress, these networks may show reduced compensatory capacity, leading to difficulties in flexible behaviour, stress regulation, or complex social interactions that may not be obvious in low-demand settings [107]. Normative-modelling and dimensional approaches are particularly well-suited to quantifying such subclinical deviations [154].

Because the model is plasticity-oriented, it generates hypotheses for intervention effects. Approaches that engage neuroplastic mechanisms such as enriched environments, structured cognitive training, aerobic exercise, and nutritional supplementation (e.g., choline) may induce measurable changes in network properties.

In animal models, environmental enrichment after PAE has been associated with improvements in spatial learning and partial normalization of hippocampal and striatal plasticity markers [154,155]. In children with FASD, choline supplementation has been reported to be associated with improved executive outcomes and possible long-term effects on corpus callosum microstructure [156,157]. Within this framework, successful interventions would be expected to be associated with increased global and local efficiency, strengthening of appropriate hub architecture, and reduced neural noise; however, the relationship between network-level changes and behavioural improvement requires further empirical validation [156,158].

Finally, animal models in which PAE is combined with targeted mechanistic manipulations provide a route to causal tests of these ideas. If antioxidant treatment, modulation of growth factor signalling, or environmental enrichment during defined developmental windows can normalize structural and functional network metrics alongside behaviour improvement, this would support a mechanistic role for these pathways in shaping emergent brain architecture [32,33,154]. Importantly, all mechanisms and predictions presented in this section are hypothesis-driven and should be interpreted with caution. They require rigorous validation through longitudinal human studies, replication across cohorts, and controlled experimental work before any translational or clinical conclusions can be drawn.

## 7. Clinical and Translational Implications

A network-level account of PAE reframes FASD as conditions in which distributed brain organization, including long-range integration, modular segregation, and hub integrity, deviates from typical neurodevelopment, rather than reflecting only isolated regional injury [121,123]. This framework aligns heterogeneous cognitive and behavioural profiles with principles of network vulnerability and developmental timing, and suggests that intervention effects may be better captured by systems-level outcomes (e.g., connectivity and efficiency) than by single-region markers. It complements established diagnostic frameworks, integrating confirmed exposure, physical features when present, and neurobehavioral impairment [159]. While this network-based framework offers valuable translational perspectives, it is important to recognize that many proposed applications remain at an early stage of development. Several network-derived measures currently function primarily as research tools, and substantial methodological validation will be required before they can be integrated into routine clinical care for individuals with FASD.

### 7.1. Early Identification and Biomarker Development

Current diagnostic pathways often rely on dysmorphology, growth indices, and neuropsychological profiles, frequently triggered when learning or behavioural difficulties become evident in school settings [159]. By that stage, atypical trajectories in large-scale organization may already be entrenched, potentially reducing the leverage of early experience-dependent plasticity [160]. A network framework therefore prioritizes earlier risk stratification, ideally in infancy or toddlerhood, so that supportive interventions can begin while neural systems remain highly malleable. However, it is important to emphasize that no network-based biomarker has yet been validated for the clinical diagnosis of FASD. Current EEG and neuroimaging-derived measures remain primarily as research tools, and their translation into routine clinical practice will require standardization of acquisition and analytical protocols, replication across independent cohorts, and demonstration of adequate diagnostic sensitivity and specificity.

Although neuroimaging and electrophysiological studies (EEG, MRI) have revealed consistent group-level alterations in PAE populations, these measures remain primarily research tools and are not yet validated for clinical diagnosis [161]. Standardization of acquisition protocols, replication across independent cohorts, and demonstration of robust diagnostic performance are required before clinical translation.

EEG offers a particularly scalable approach for probing early brain function, with studies reporting alterations in neonatal and childhood activity patterns in exposed cohorts [160,161]. Complementary measures, including resting-state spectral features, connectivity indices, and event-related potentials, provide developmentally appropriate windows into network organization [162]. These approaches are most promising when integrated into multimodal risk models combining clinical exposure history, developmental trajectories, and emerging biological markers. At present, they should be considered exploratory tools for research and risk stratification rather than established screening methods.

### 7.2. Multicomponent, Network-Informed Interventions

Because PAE affects multiple functional domains, single-modality interventions are unlikely to address the full phenotype [163]. A network perspective supports multicomponent interventions targeting cognition, behaviour, language, motor function, and environmental structure.

Such approaches aim not only to build compensatory strategies but also to engage distributed neural circuits through structured developmental experience [121,123].

Combined cognitive training, behavioural support, and enriched environmental input may enhance recruitment of executive networks and reduce cognitive load [162,163]. Network-sensitive outcomes (EEG, MRI) may be used in research contexts to examine whether behavioural improvements are accompanied by changes in neural organization. However, their clinical significance and responsiveness remain uncertain [164].

### 7.3. Expectation Management and Lifespan Support

Conceptualizing FASD as a disorder of distributed network function can improve communication with families, educators, and clinicians by clarifying why difficulties may appear inconsistent across contexts and why performance often deteriorates under stress or increased cognitive load [147]. This framing helps reduce misattributions (e.g., lack of effort or “bad behaviour”) and supports realistic, compassionate expectations.

Equally important is a lifespan-oriented care model. Many individuals will benefit from sustained supports that adapt to predictable transitions such as entry into secondary school, vocational training, independent living, or workforce participation when executive and social cognitive demands typically intensify. A network lens also encourages a strengths-based approach: identifying relatively preserved capacities and building interventions around these assets can improve engagement, self-efficacy, and quality of life.

### 7.4. Neurotechnology: Opportunities and Cautions

Neuromodulation and neurotechnology tools (e.g., neurofeedback, transcranial direct current stimulation [tDCS], transcranial magnetic stimulation [TMS]) are increasingly explored in pediatric neurodevelopmental conditions. In principle, these approaches could be leveraged in FASD to alter excitability and facilitate recruitment of underperforming networks, particularly when paired with cognitive training that provides a behavioural target for plastic change. A sham-controlled randomized trial combining tDCS with cognitive training in children with FASD illustrates both translational promise and the need for careful evaluation [165]. Importantly, the current evidence base remains extremely limited, with relatively few studies conducted specifically in individuals with FASD and with modest sample sizes. Consequently, no neuromodulatory intervention can presently be recommended for routine clinical use in this population.

However, the diffuse and developmentally evolving nature of network alterations in FASD necessitates caution. Potential risks include reinforcing maladaptive activation patterns, disrupting compensatory pathways, or producing age-dependent effects that differ from adult protocols. Translation therefore requires conservative dose finding, developmentally tailored stimulation parameters, rigorous sham-controlled designs, and close monitoring of benefits and unintended effects. At present, neurotechnology approaches should be regarded as experimental. Their use should remain confined to rigorously designed research protocols until their safety, efficacy, optimal stimulation parameters, and long-term effects have been established in adequately powered clinical trials.

### 7.5. Strengthening Prevention Efforts

Finally, a network-based model has direct public health relevance. By emphasizing that even “moderate” PAE can reshape neural system organization rather than producing only isolated deficits, health professionals may communicate risk more persuasively. Public-health guidance remains clear that there is no known safe amount, no safe time, and no safe type of alcohol use during pregnancy [166]. Effective prevention must therefore combine clear abstinence messaging with practical supports for women of childbearing age, including accessible screening, non-stigmatizing counselling, timely referral to treatment for alcohol use disorders, psychosocial resources, and reliable contraception options [167].

Testing and refining the neuro-computational architecture model of PAE will require coordinated, interdisciplinary research. Longitudinal multimodal cohort studies are crucial. Prospective cohorts of infants with documented PAE, followed into adolescence with repeated structural and functional MRI, diffusion imaging, EEG/MEG, and detailed neuropsychological assessments, would allow direct observation of how network trajectories diverge from typical development, which network features best predict outcomes, and when critical divergence points occur [168].

Integration of omics data, genomics, epigenomics, transcriptomics, and metabolomics with network metrics can identify molecular signatures associated with particular network phenotypes. For example, specific DNA methylation patterns at neurodevelopmental genes might predict altered connectivity in fronto–limbic circuits or reduced myelin integrity in long association tracts.

Computational modelling of developing networks offers a complementary approach. Simulations in which nodes grow, form connections, and undergo activity-dependent refinement under different perturbation regimes (e.g., increased apoptosis at specific times, altered activity patterns, weakened trophic signals) can generate predictions about network topology and dynamics that can then be compared with empirical data.

Experimental work in animal models can identify critical windows for intervention by varying the timing and type of postexposure interventions (environmental enrichment, pharmacological agents, growth factor supplementation, antioxidant treatments) and assessing their effects on network metrics and behaviour. Translation of these findings to human development needs to consider species differences in timing but can offer valuable guidance.

Finally, there is a need to develop and validate practical, scalable biomarkers for clinical and public health use, such as simplified EEG protocols, eye tracking paradigms indexing network-level processing, or autonomic markers that correlate with central network organization. These tools should be evaluated for their predictive value in diverse populations and settings. Despite the substantial body of evidence reviewed herein, several limitations of the current literature should be acknowledged. Many studies investigating the neurodevelopmental consequences of PAE are potentially influenced by several confounding factors. Prenatal tobacco exposure, maternal stress, socioeconomic adversity, nutritional deficiencies, co-exposure to other substances, and postnatal environmental conditions may independently affect brain development and behavioural outcomes. Consequently, disentangling the specific contribution of alcohol from these interacting factors remains challenging, particularly in human studies. These potential confounders should be considered when interpreting findings from the current literature and represent an important limitation of the field.

In addition to these confounding factors, the neuroimaging and connectomic literature in PAE is itself characterized by considerable methodological heterogeneity, including variable imaging protocols, parcellation schemes, connectivity reconstruction methods, and analytic thresholds, which complicates direct comparison across studies. Many individual findings, particularly at the level of specific network metrics or hub regions, await independent replication in adequately powered, harmonized cohorts, and this reproducibility gap should be kept in mind when interpreting the overall coherence of the systems-level evidence presented above.

## 8. Conclusions

PAE exerts multifaceted effects on the developing brain, spanning cell death, oxidative damage, disrupted signalling, glial dysfunction, and epigenetic reprogramming. These mechanisms, acting during critical windows, reshape the trajectory of brain development. Structural and functional neuroimaging and electrophysiology indicate that the consequences are not confined to focal lesions or simple volume loss but involve pervasive alterations in how the brain’s networks are wired and dynamically coordinated.

Conceptualizing FASD as a disorder of emerging neuro-computational architecture provides a useful integrative framework connecting molecular and cellular events to systems-level organization and clinical phenotype. This may help to account for why FASD affects multiple cognitive and behavioural domains, why deficits often emerge or worsen with age as demands on network integration increase, and why interventions that broadly enhance plasticity and environmental support can be effective despite the absence of a single “target lesion.” This framework does not diminish the importance of classical mechanisms; rather, it integrates them into a systems-level perspective. It emphasizes both vulnerability and plasticity: PAE can irrevocably alter some aspects of brain wiring, but the developing brain retains an impressive capacity for compensation and reorganization. By leveraging this capacity through early identification, enriched environments, and network-informed interventions, it is possible to improve outcomes for individuals with FASD.

Importantly, the concept of FASD as a disorder of emerging neuro-computational architecture should currently be regarded as a promising conceptual and integrative hypothesis rather than a directly validated model. Although converging evidence from molecular, cellular, neuroimaging, electrophysiological, and behavioural studies supports this perspective, this evidence is at present largely correlational and consistent with, rather than confirmatory of, the framework. Further empirical research is needed to establish causal links across these levels of organization and to test the specific predictions generated by this framework. Future studies combining mechanistic, longitudinal, and network-based approaches will be essential to determine its explanatory and translational value.

From a societal perspective, preventing PAE remains an urgent priority. At the same time, for the many individuals already affected, a deeper understanding of how their brains are wired can foster more informed, compassionate, and effective support, transforming a story of disrupted connections into one of adaptive re-wiring and resilience.

## Data Availability

No new data were created.
